# To Taste or Not to Taste: A Narrative Review of the Effectiveness of Taste and Non-Taste Exposures on the Dietary Intake of Head Start Children

**DOI:** 10.3390/nu17111817

**Published:** 2025-05-27

**Authors:** Anna R. Johnson, Nathaniel Richard Johnson

**Affiliations:** Department of Nutrition and Dietetics, University of North Dakota, Grand Forks, ND 58202, USA; anna.johnson.1@ndus.edu

**Keywords:** food exposure, head start, nutrition education, willingness to try food, dietary intake in children

## Abstract

**Objectives**: Limited variety in children’s diets impairs lifelong nutrition and health. Head Start is a federal program serving expectant families and children in the United States living at or below the poverty line to the age of five. Head Start children face barriers to nutrient intake. Many nutrition education curricula are implemented in Head Start settings; however, few have addressed whether taste or non-taste food exposures are more effective and appropriate for improving dietary intake in this population. This review evaluates if taste or non-taste exposures are more effective at increasing willingness to try, consume, and like food in children participating in Head Start. **Methods**: PubMed was searched for studies published in the last 10 years with children aged 2 to 12 years. Included studies had an intervention with exposure to food or its likeness, focusing on those studying Head Start or similar samples. Articles were excluded if they referenced exposure to marketing, disease, or foodborne illness. **Results**: Searches yielded 903 results. 51 articles were screened, and 15 were included in the narrative. Studies revealed that combinations of taste and non-taste exposures improved children’s willingness to try, consume, and like food. **Conclusions**: Taste and non-taste exposures, when used independently, inconsistently affect children’s willingness to try, consume, and like food; exposures are most effective when combined, although research on the topic faces limitations of study design and environmental controls. With federal standards for nutrition, Head Start programs should implement food exposure activities. Additional studies with improved designs and controls for exposure to the environment should be completed in this population to increase the validity and reliability of food exposure research.

## 1. Introduction

A nutritious diet for infants and children is critical for proper growth and development while setting the trajectory for lifelong eating habits and health [1]. The 2020–2025 version of the Dietary Guidelines for Americans recommends a healthy dietary pattern including fruits, vegetables, whole grains, dairy, and protein at all ages and stages [1]. Despite recommendations, many children do not consume fruits and vegetables daily, which is exacerbated by income, with children living below the federal poverty level consuming less fruit and a decreased variety of vegetables, specifically dark green vegetables [2]. Limited intake in children may have lasting impacts on nutrition and development [3,4].

Head Start, a federal program serving expectant families and children to the age of five, supports families in overcoming barriers and improving their child’s nutrition [5]. More specifically, this no-cost, nationwide program promotes development and school readiness for children and families living below the federal poverty line. Head Start programs must follow the Head Start Act and Head Start Program Performance Standards (HSPPS), designated in part 1302 of the Code of Federal Regulations [6]. Parts 1302.44 and 1302.46 of the HSPPS include requirements for nutrition. Programs must provide culturally and developmentally appropriate nutrition services that follow the Child and Adult Care Food Program, a child nutrition program of the United States Department of Agriculture. Additionally, programs must assess each child’s nutrition status and work with caregivers to support children’s nutrition. With these requirements, Head Start programs provide an appropriate environment for implementing nutrition education.

Children experience dietary intake changes as they grow and develop; some of these changes are considered normal, whereas others concern parents [7]. This is especially true for intakes of vegetables and new foods due to food fussiness, leading to the rejection of new and familiar foods [7,8,9]. For instance, Brown and colleagues reported similar food fussiness scores across Head Start children of various races, ethnicities, body mass indexes, and food security levels [9]. Despite expert recommendations, many children do not eat a variety of nutritious foods, as children experience barriers to intake, including food fussiness [1,2]. Children are exposed to food and flavors in various ways, beginning in utero, creating a sense of familiarity. Over 50 years ago, Zajonc coined the term, mere exposure, stating, “mere repeated exposure of the individual to a stimulus is a sufficient condition for the enhancement of his attitude toward it” [10] (p. 1). Although Zajonc’s mere exposure initially applied to word frequency, the concept applies to dietary intake as well, with examples ranging from research on the effects of exposure on portion size, vegetable flavor exposure in early feeding, and optimal service frequency of vegetables to children [11,12,13]. Thus, mere exposure can be applied to children’s diets in an effort to address food fussiness and improve dietary intake.

Applying mere exposure in children’s diets can involve interactions with food or its likeness, referred to as taste or non-taste exposure in this manuscript. In this review, taste exposures are repeated opportunities to put food into the mouth. Non-taste exposures include tactile, olfactory, and visual sensory interactions with food or likeness without the pressure to taste, such as a picture, story, game, craft, gardening, or cooking. Taste and non-taste exposures can also be offered in a mixed methods approach.

In fact, due to the HSPPS requirements, Head Start is an ideal environment to implement food exposures to improve dietary intake [6]. With the many nutrition education curricula implemented in Head Start settings, few address the effectiveness of the exposure methods often incorporated in the program. This narrative review aims to compare and evaluate research on food exposures on children’s dietary intake. More specifically, this review evaluates whether taste or non-taste exposures are more effective at improving dietary intake by increasing willingness to try, consume, and like food in Head Start preschool children.

## 2. Methods

Search terms included combinations of the following: food exposures, exposures, repeated exposures, repeated tasting, taste-exposures, non-taste exposure, food, food neophobia, mealtime, non-mealtime, gardening, United States, Head Start, preschool, WIC, food insecurity, low income, children, and willingness to try. Searches were completed in the PubMed database between October 2024 and February 2025, filtering for articles published in the last 10 years on children between the ages of two and 12 years of age. In addition, the reference lists of relevant articles were used to review related studies.

Articles were screened for inclusion and exclusion criteria, included in Table 1. Included studies focused on a sample of low-income families and children between 12 months and 5 years of age in the United States, such as those enrolled in Head Start or other assistance programs. However, wider economic and geographic samples were included if research within Head Start centers was limited. Included articles provided an intervention that involved an interaction with food or its likeness and presented results regarding willingness to try foods, amounts of foods consumed, and/or the likability of foods. Studies referencing exposure to marketing, disease, or foodborne illness were excluded.

## 3. Results

**Figure 1:**
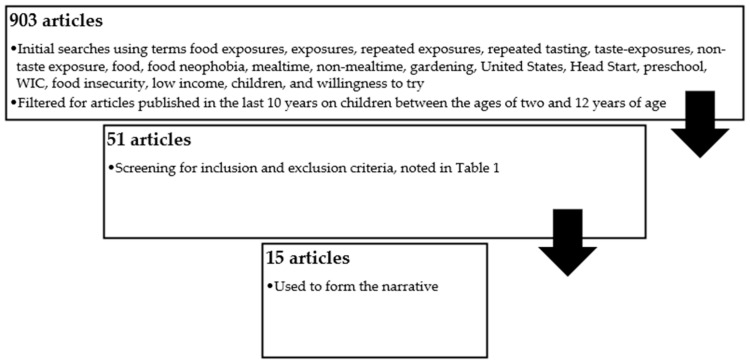
Flow diagram of narrative review process.

Interacting with food in educational settings provides opportunities to learn about eating and culture while connecting school to home life [14]. Interactions with food or its likeness can involve exposing children to food with or without the pressure to taste, referred to as taste or non-taste exposure in this work. Taste exposures are repeated opportunities to put food into the mouth. Non-taste exposures include interactions with a food likeness, such as a picture, story, game, or craft; additionally, non-taste exposures include activities with real food without the pressure to eat, such as when gardening or cooking. Non-taste exposures can be visual, tactile, or olfactory [15]. Many nutrition education programs in preschool settings implement a mixed methods approach using both taste and non-taste exposures. Research on this topic was completed using the previously described methods, depicted in Figure 1, resulting in a total of 903 articles. Of these, 51 articles were reviewed, and 15 were used to form the narrative. A synopsis of the 15 studies is presented in Table 2.

### 3.1. Taste Exposures

Regular taste exposures are often provided through meal services in preschool settings. A study by Izumi and associates implemented an eight-month intervention adding targeted fruits and vegetables to Head Start menus twice weekly [18]. A center not receiving repeated exposures was compared using a multilevel logistics model for nested data. Repeated taste exposures created a significant effect on willingness to try three of nine target foods (turnip OR = 3.5, 95% CI [1.5–9.2], rutabaga OR = 3.9, 95% CI [1.7–9.1], and beet OR = 2.4, 95% CI [1.1–5.4]) compared to the control group; no significant difference was found in liking any of the vegetables compared to the control. A study by Nekitsing and colleagues also assessed the effects of taste exposure in preschool children [22]. This United Kingdom-based intervention served mooli, a daikon variety, as a weekly snack. After the 10-week intervention, mooli intake increased, which continued through two follow-up test points at 24 and 36 weeks (intake of eaters *F*_(1, 135)_ = 11.21, *p* = 0.001). The intake of those not receiving the repeated taste intervention increased at smaller intervals at each data collection point. Additionally, the percentage of children willing to taste mooli increased in the taste exposure group, albeit insignificantly, whereas the comparison group remained steady throughout the intervention. Taste exposure can increase preschool children’s willingness to try food and consumption levels [18,22].

### 3.2. Non-Taste Exposures

Studies focusing on non-taste sensory experiences in preschool-aged populations improve their willingness to interact with food. Roberts and colleagues compared south-east England nursery children’s exposures to six vegetables (broccoli, fennel, leek, parsnip, radish, and swede) using a variety of visual, tactile, and olfactory sensory experiences [15]. Non-taste exposures were provided using colored posters, fabric, and lids to control the sensory experience. Exposures targeting multiple senses have the greatest positive effects on willingness to try (U = 171.0, *p* < 0.001, η^2^ = 0.26) and increase liking (U = 193.0, *p* = 0.001, η^2^ = 0.19). Dazeley and Houston-Price assessed willingness to try in a non-taste exposure study targeting visual, olfactory, tactile, and auditory sensory experiences in United Kingdom nurseries [17]. This study found an increase in children’s willingness to engage with food; while the increase in willingness to taste was not significant, a significant increase was experienced in children’s willingness to touch (t(53) = 2.05, *p* = 0.046), leading to promising possibilities of future tasting. Sensory play with food or its likeness improves children’s willingness to engage with foods.

Nutrition education programs can contain non-taste exposure experiences, such as the Phunky Foods program, used in the study by Nekitsing and colleagues referenced in the taste exposure section [22]. Phunky Foods combined visual, auditory, and tactile activities with education on nutritious eating, but did not explicitly include the target vegetable, mooli. This program was implemented independently in an intervention group, with increased mooli intake throughout the study, although the increase was smaller than the taste exposure intervention. The number of children unwilling to try the mooli decreased from pre-intervention to post-intervention, with a small increase by the 36-week follow-up. Non-taste sensory exploration benefits children’s diets when included with nutrition education, although this study and the generalization of effects are limited by minimal data analysis.

Research on visual food exposures suggests minor dietary improvements. Caputi and researchers developed a set of e-books focusing on vegetables from farm to fork, aiming to provide a positive interaction between adults and children while reading about food [8]. This two-week study focused on Italian children aged 18–48 months. Despite increases in target and control vegetables, as well as significant main effects for willingness to try (*F*_(1, 48)_ = 15.06, *p* < 0.001, ηp^2^ = 0.239), portion size (*F*_(1, 48)_ = 23.80, *p* < 0.001, ηp^2^ = 0.332), consumption frequency (*F*_(1, 46)_ = 8.47, *p* = 0.006, ηp^2^ = 0.156), and liking (*F*_(1, 48)_ = 22.04, *p* < 0.001, ηp^2^ = 0.315), no significant interaction was found between vegetable and time for any variable. Masento and team completed a similar study using “See and Eat” e-books in the United Kingdom [20]. With identical procedures, this study did find significant interaction effects between the condition and time for portion size (*F*_(1,29)_ = 15.55, *p* < 0.001, ηp^2^ = 0.349), consumption frequency (*F*_(1,30)_ = 13.49, *p* = 0.001, ηp^2^ = 0.31), and liking (*F*_(1, 29)_ = 8.69, *p* < 0.05, ηp^2^ = 0.231). While small in both studies, intake of the control vegetable did increase, indicating general dietary benefits. Masento and colleagues found no significant associations between any demographic indicator and measures of change in the target vegetable, indicating non-taste, visual food exposure benefited children regardless of background [20]. Visual exposures can positively influence children’s dietary choices.

Emerging research on gardening as a nutrition intervention for preschool-aged children also shows promise. A study by Monsur and colleagues assessed food liking after a gardening intervention at eight centers in Texas [21]. The garden was implemented and an instructional book guided 12 classroom activities on produce preparation, maintenance, and harvest. The children rated 12 fruits and vegetables on a five-point scale, with a significant increase from pre- to post-intervention in combined fruit and vegetable rating (est. effect = 1.20, *p* = 0.01) and vegetable scores (est. effect = 2.05, *p* = 0.04) when controlling for gender, age, and ethnicity; however, this study had limited participant data and significant differences in group age and ethnicity. Although studies are limited on preschool-aged children, gardening, like visual exposures, has potential as a powerful non-taste exposure tool.

### 3.3. Mixed Method Exposures

Various preschool nutrition education programs utilize mixed-method exposures. Combining taste and non-taste exposures creates well-rounded opportunities for children to interact with food without the expectation of eating, coupled with opportunities to taste food. The Harvest for Healthy Kids curriculum includes books, tasting activities, sensory table ideas, crafts, picture cards, and newsletters [18,24]. Izumi and colleagues combined the program with additional exposures discussed in the taste exposure section of this paper [18]. The Food Friends-Fun with New Foods^®^ program introduced foods with characters and activities over a 12-week program in the Colorado LEAP study [19]. Non-taste exposure activities in this program included puppet shows, activity cards, books, songs, art projects, sorting, and games, while taste exposures were included as snacks. The Together, We Inspire Smart Eating (WISE) program uses a barn owl mascot, Windy Wise, a barn owl, to engage children in food and nutrition [25,26]. The curriculum incorporates food preparation experiences, integration of food into educational activities, family handouts, and interactions via social media. Lastly, the Phunky Foods program, mentioned in the non-taste exposure section, implements hands-on activities from two components, Eat Well and Strive for 5! [22]. Many preschool nutrition education curriculums combine exposure experiences to enhance children’s nutrition.

Combining exposures offers a successful strategy for increasing children’s willingness to try food. The combined intervention completed by Izumi and colleagues boosted willingness to try, creating a significant increase in six foods (carrot OR = 5.7, CI [1.4–23.0], butternut squash OR = 2.9, CI [1.1–7.4], sweet potato OR = 4.3, CI [1.8–10.7], turnip OR = 7.2, CI [2.7–10.5], rutabaga OR = 9.6, CI [3.8–24.5], beet OR = 6.1, CI [2.3–16.3]) compared to three in the foodservice intervention group discussed in the taste exposure section of this paper [18]. While a significant difference developed between the comparison and the combined intervention, no significant difference existed between the combined intervention and the foodservice-only intervention; however, this lack of significance may be due to the limited sample size [18]. The study referenced in the taste and non-taste sections by Nekitsing and researchers measured the number of children who did not eat the mooli at four data collection points, although provided minimal data analysis in reporting [22]. In the mixed-method intervention, the number of children who did not eat the vegetable decreased from pre-intervention to post-intervention, with a slight increase at a 24-week follow-up, it returned to post-intervention levels by 36 weeks. Combining exposure methods can strengthen effects on children’s willingness to try.

Mixed method exposures can increase children’s positive feelings toward food. While the foodservice intervention by Izumi and researchers discussed in the taste exposure section did not significantly impact liking, the combined intervention did result in a significantly higher score for sweet potato (OR = 3.4, 95% CI [1.2–9.5]), turnip (OR = 3.7, 95% CI [1.2–11.2]), and berries (OR = 21.2, 95% CI [2.4–187.3]) when compared to the control group with a multilevel logistics model [18]. A pilot study in Head Start Centers by Schmitt and colleagues showed mixed method exposure lessons and play led to a statistically significant increase in vegetable liking (M change = 1.1, *p* = 0.02), while the comparison group did not experience a significant change [23]. In the Colorado LEAP study, the percentage of positive ratings increased at each data collection point; however, the intervention and control group increased at consistent rates and the difference between groups did not reach statistical significance [19]. Data suggests combined exposure methods can increase liking, although liking of food may increase naturally with development.

Consumption increases when children receive mixed-method exposures. In the Colorado LEAP study, the intake of the target vegetable jicama in the intervention group increased significantly from baseline to post-intervention (M = 34.1 g, CI [28.2–40.0], *p* < 0.0001) and remained elevated at the two-year follow-up (M = 37.3 g, CI [30.8–43.8], *p* < 0.0001) [19]. The comparison group did not experience a significant change post-intervention but increased significantly at follow-up (M = 20.3 g, CI [12.6–28], *p* = 0.0008). The assessment included a second food without exposure, edamame, with similar average intakes for both groups at baseline. By the two-year follow-up, both groups reported significantly increased edamame intake (intervention M = 20.8 g, CI [12.6–29.1], *p* < 0.0001; comparison M = 28.9 g, CI [19.9–37], *p* < 0.0001) despite the comparison group not experiencing a significant change post-intervention. Differences in intake between jicama and edamame at two years illustrate the importance of exposures on increasing intake, and the role development plays in dietary intake. Nekitsing and colleagues, previously mentioned in the taste and non-taste sections, combined exposures using the Phunky Foods program and compared mean intakes with limited data analysis [22]. Intake increased at post-intervention and the 24-week follow-up but decreased slightly at the 36-week follow-up, compared to peak consumption at 24 weeks. Without consistent and frequent exposures throughout development, effects may plateau, leading to consumption similar to children without exposure; however, children will benefit from the nutrients consumed during the increase.

## 4. Discussion

This narrative review aims to evaluate whether taste or non-taste exposures are more effective at improving dietary intake in Head Start children by increasing their willingness to try, the amount consumed, and the likability of food. Implementing food exposures into Head Start environments benefits children’s diets, as evidenced by the overview of exposures in Table 3. Foodservice and teaching staff can establish a cohesive approach to provide consistent taste and non-taste exposures. As there is flexibility in nutrition standards from the Head Start Program Performance Standards (HSPPS), programs can tailor their approach to their community’s needs. The HSPPS encourage community partnerships, and programs could work with local nutrition experts, such as Extension, to implement a researched curriculum [6]. Additionally, programs must employ or consult a registered dietitian or nutritionist to support the nutritional aspects of the program, which could include designing or implementing food exposure activities [6]. When executing exposure efforts, programs should consider how to include caregivers and the unique needs faced by the population.

Including caregivers is pivotal to success with dietary changes, as they control children’s diets at home. In a focus group of parents with children in daycare centers in French cities, children’s picky eating concerned and frustrated parents [7]. All but one of the 38 parents reported changes in their child’s diet, with a majority experiencing this around age two. “He used to eat everything and overnight he started to be difficult”, was a common theme in focus groups [7] (p. 407), highlighting that children’s liking of foods changes as they develop. Some parents understood the importance of taste exposures for improving dietary intake, but more could be done to reinforce food exposures. On that note, one parent stated, “Clearly if there is something she doesn’t like, I have to offer it again over the following weeks until she eats it” [7] (p. 409). Parents understand the importance of creating positive exposures to increase familiarity, with some creating characters or serving condiments with new foods. Studies addressing food exposures highlight the causal effect of increased familiarity with food, a critical intervention as growing children learn to assert their independence [7,8,15,17,20,22]. With Head Start serving children at the age when dietary changes are commonly reported, an exposure intervention could be implemented during programming and made more effective by providing support for caregivers at home.

The effects of a school-based intervention can be seen in the home diet and could be explained by children’s pester power. Studies by Whiteside-Mansell and Swindle and Swindle and colleagues assessed the at-home effects of the Together, We Inspire Smart Eating (WISE) nutrition program, using a character, Windy Wise, and sensory experiences to expose children to foods in a Head Start classroom [25,26]. The intervention group experienced an increased home intake of all food categories, except sugary sweets, which decreased [26]. The comparison experienced smaller increases, including an increase in the sugary sweets category, and a decrease in consumption of three WISE fruits targeted in the program: apples, strawberries, and blueberries. A regression analysis controlling for demographics and pre-intervention consumption showed a statistically significant positive effect in the dark green, orange, and yellow vegetable category (SE = 0.050, t = 2.12, *p* < 0.05) and WISE fruits (SE = 0.050, t = 2.12, *p* < 0.05), while sugary sweets intake experienced a significant negative effect (SE = 0.050, t = −3.27, *p* < 0.01). Fruits in general and the targeted WISE vegetables (tomatoes, sweet potatoes, carrots, bell peppers, spinach, greens, and green beans) did not have a significant effect. These at-home effects may be explained through the power of children’s pestering. Swindle and colleagues used hierarchical regression analysis to demonstrate the effect children’s pester power can have on the home diet after a nutrition education program [25]. Controlling for pretest values, willingness to try, and location effects, pester power was a significant predictor of targeted WISE fruit and vegetable consumption (B = 0.16, β = 0.29, *p* < 0.01), but not of nutrient-poor food intake. Overall, positive changes to the home diet occur after a classroom-based nutrition education program and efforts should be made in supporting positive pester power to amplify effects.

Considerations, such as food waste and accessibility, must be made when working with populations living below the federal poverty line, including Head Start enrollees. Taste exposure effects may take weeks to develop, as demonstrated by the eight-month intervention by Izumi and associates and the 10-week intervention by Nekitsing and colleagues, leading to food waste when repeated exposures are served despite children’s unwillingness to try [18,22]. E-books, gardening, and non-taste sensory experiences revealed positive effects on willingness to try, showing promise as a budget- and environmentally friendly alternative to food wasted from repeated taste exposures [8,15,20,21,22]. With proper attention to the technical challenges of users, device accessibility, and storage space, technology can be leveraged to increase access with a simple and cost-effective electronic distribution process [8,20].

Considerations of nutrition programs for Head Start families extend to food access. A study of rural Head Start children assessed blood carotenoid levels in three groups with the control receiving regular Head Start services, treatment A receiving high-carotenoid fruits and vegetables weekly, and treatment B receiving weekly food and the previously discussed Harvest for Healthy Kids program, including both taste and non-taste exposures [24]. Blood carotenoid levels were assessed using a Pharmanex Biophotonic S3 Scanner using Resonance Ramen Spectroscopy. All three groups experienced an increase in mean carotenoid scores, led by treatment B, with statistically significant differences between treatments (*F*_(2, 206)_ = 12.967, *p* < 0.001, Cohen D = 71), demonstrating the causal effects of access and exposures. Considerations for decreasing waste, program delivery, and access to nutritious food can create a more inclusive and effective exposure program.

Various factors limit the ability to draw conclusions from research on food exposures. Differences in study design, small sample sizes, and self-reported measures limit validity and reliability. Moreover, the implementation of nutrition education curricula is limited by staff and the environment. The studies on e-book exposures provide examples of small sample sizes used in exposure studies, with the Italian sample reporting an underpowered sample for evaluating intake [8,20]. Many studies were non-randomized, using a quasi-experimental design, such as the non-randomization of the comparison group in the Harvest for Healthy Kids pilot study [18]. The long-term effects of the Colorado LEAP study were obscured by unclear longitudinal data collection [19]. Additional limitations include recency bias, demographic differences, limited statistical analysis, and high attrition; the Harvest for Healthy Kids study by Izumi and researchers included only 49% of the sample in the analysis due to Head Start enrollment changes, and the study by Monsur and colleagues faced statistical differences in age and race in the control and experimental groups [18,21]. Dixon and colleagues reported fluctuations in liking and eating, with one teacher stating children “will eat carrots one day but not the next” demonstrating the variability and challenges of consistently measuring child data [14] (p. 12). Despite this bias, many studies utilized child- or parent-reported values. Overall, studies on food exposures had factors limiting validity and reliability.

Future research could improve study designs and measurement techniques, while controlling potential environmental influences. While six studies utilized trained observation or weight to measure willingness to try and intake levels, Smith and Colleagues provide a gold standard for measuring consumption [15,16,17,18,19,22,24]. By using spectroscopy to measure blood carotenoid levels, dietary intake was objectively assessed using unintrusive technology, providing more effective measures of program success than child- or parent-reported intake or observation, where errors may occur [24]. Additionally, this study could be used as a design model as there are limited experimental studies on food exposures, and it boasted 0% attrition [24].

Positive initial experiences may lead to more immediate exposure benefits. In the Colorado LEAP study, children rating jicama more positively at baseline had larger increases in consumption during follow-up [19]. Those in the intervention group who initially rated jicama as yummy or just OK experienced the most significant increase in grams consumed, with the increase continuing to the two-year follow-up for those rating jicama as yummy (mean change = 21.4 g CI [13.4–29.5], *p* < 0.0001). Children rating jicama as yummy or just ok in the control group approached a significant increase in grams consumed during the two-year follow-up. Immediate reactions influence exposure effectiveness, making the first interaction critical to success.

The intervention environment is vital to positive first impressions; however, exposure studies did not reference or control for staff or environmental influences. A cross-sectional study by Anundson and colleagues observed the effects of staff behaviors on children’s eating habits, demonstrating the causal impact of adults on children’s diets [16]. When staff sat with children and ate the same food, vegetable tasting increased significantly (mean difference = 1.02, *p* < 0.05) while tasting of high-fat/high-sugar foods decreased at a rate approaching significance. Adults sitting at the table without eating the same food significantly decreased fruit tasting (mean difference = −1.24, *p* < 0.05), with smaller impacts on other food groups. When children were exposed to healthy food talk, tasting fruits increased significantly (mean difference = 0.92, *p* < 0.05), and tasting high-fat/high-sugar foods decreased at the highest rate (mean difference = −0.65, *p* < 0.001). Lastly, staff checking in with children before finishing a meal demonstrated significant increases in fruit tasting (mean difference = 1.31, *p* < 0.001), while decreasing tasting fried vegetables, fried meat, and high-fat meat, and the most significant decrease in high-fat/high-sugar foods (mean difference = −0.69, *p* < 0.001). Staff behaviors, as part of the classroom environment, influence children’s nutrition-related behaviors.

Despite influencing children’s diets, educators may not possess the ability to execute nutrition-based learning or interventions. This was demonstrated in the taste exposure study completed in the United Kingdom where staff did not follow the taste exposure protocol, decreasing the reliability and validity of the results [22]. In a qualitative study by Dixon and colleagues, teachers reported discomfort in implementing food-based learning, or incorporating learning with food into meals, science, and other educational concepts; education at meals comes more naturally to teachers than implementing food-based learning during other class times [14]. Teachers often misunderstand the intention of using food in learning, choosing foods children are comfortable with or calorie-dense items. Additionally, many Head Start programs, with supportive intentions, write policies prohibiting or limiting the use of real food; Head Start families may experience food insecurity, and programs feel it is inappropriate to use food in lessons without a plan to consume [14]. Short training sessions were provided before implementing many nutrition curricula, but the limitation of overcoming prior values, beliefs, and knowledge still exists.

Future studies on food exposure effectiveness could measure or control for staff and environmental impact. Teachers reported being uncomfortable with nutrition concepts and may not understand what pressure, reward, or withholding food looks like in practice, negatively affecting the intentions of a food exposure intervention [14]. Lack of control or measurement of these variances may skew an exposure intervention. Teachers may also be uncomfortable or unaware of other concerns regarding food-based learning, including proper sanitation, safe preparation, and special dietary needs [14]. Due to teachers’ unfamiliarity with nutrition education, measuring or controlling for adult behaviors in an exposure study will provide data on intervention fidelity.

## 5. Conclusions

Despite recommendations to consume a variety of foods, children’s diets often fall short, impacting long-term health and nutrition habits. Children living below the federal poverty line experience barriers to intake, further limiting diet variety. However, this narrative review demonstrates the positive influence of mere exposure to food, or its likeness, on children’s diets. Taste and non-taste exposures, when used independently, inconsistently affect children’s willingness to try, consume, and like food; exposures are most effective when combined, although research on the topic faces limitations of study design and environmental controls. This review found that mixed food exposures, often provided in preschool nutrition education curriculums, significantly improve children’s willingness to try, consume, and like food. Head Start programs can include food in learning with considerations to program access and food insecurity; for example, when using food, programs can ensure a plan is in place for consumption or programs can include non-taste exposures with taste exposures from regular meal services. Combining the knowledge of the positive effects found in this review with Head Start standards for meal service and nutrition education, the authors conclude that Head Start classrooms provide an ideal setting for implementing and collaborating with caregivers on food exposure activities; however, due to limitations, this review proposes the need for food exposure studies on Head Start samples with increased validity and reliability, including using new technology to measure impact, implementing an experimental design, and controlling influences from the environment. Although findings were limited, children in Head Start may benefit from programs implementing exposure activities, and expanded research focusing singularly on food exposures could lead to stronger recommendations for widespread implementation.

## Figures and Tables

**Table 1:** Summary of inclusion and exclusion criteria.

Criteria	Defined
Inclusion	Preschool-aged children, intervention with food or a likeness, willingness to try, amounts of food consumed, likability of food, written in English
Exclusion	Marketing, disease, foodborne illness, children in elementary school or above, infants, non-English manuscripts
Filters	Children between the age of 2–12, articles published in the last 10 years
Participant Characteristics	Children aged 12 months to 5 years, Head Start participants preferred and supplemented with external populations when Head Start research was limited

**Table 2:** Highlights of exposure research.

Reference	Study Design, Exposure Type	Population	Intervention or Comparison	Methods	Results
[16] Anundson et al., 2018	Cross-sectional assessing staff impact on taste exposure	Three- to five-year-old children enrolled in 25 Oklahoma childcare centers (*n* = 201), including two Head Start and 11 tribally affiliated centers	Classroom observations were conducted to assess associations between staff’s food behaviors and children’s tasting. Behaviors were categorized as internal satiety cues, modeling, external satiety cues, and general. The behaviors were assessed with children’s tasting of food to determine the influence of staff behavior on tasting.	Researchers collected data via observation. Staff behaviors were measured with the Environmental and Policy Assessment Observation Instrument. The Dietary Observation for Child Care method was chosen to assess child tasting through plate waste.	Tasting with staff checking in with children before finishing a meal:
					Fruit mean difference = 1.31, *p* < 0.001 High-fat/high-sugar food mean difference = −0.69, *p* < 0.001
					Tasting with staff sitting at the table without eating the same food:
					Fruit mean difference = −1.24, *p* < 0.05 High-fat meat mean difference = −0.61, *p* < 0.05
					Tasting with staff sitting with children and eating the same food:
					Vegetable mean difference = 1.02, *p* < 0.05
					Tasting with staff using food to reward:
					Fruit mean difference = −2.40, *p* < 0.001 High-fat meat mean difference = −0.50, *p* < 0.05 High-fat/high-sugar mean difference = −0.74, *p* < 0.001
					Tasting with staff using food to control:
					Fruit mean difference = 0.92, *p* < 0.05 Low-fat dairy mean difference = 0.17, *p* < 0.05
					Tasting with staff encouraging to try a bite:
					Fruit mean difference = −2.18, *p* < 0.001 Fried vegetable mean difference = −0.70, *p* < 0.05 Fried meat mean difference = −1.11, *p* < 0.05 High-fat meat mean difference = 0.65, *p* < 0.001
					Tasting with staff using healthy food talk:
					Fruit mean difference = 0.92, *p* < 0.05 High-fat/high-sugar food mean difference = −0.65, *p* < 0.001
[8] Caputi et al., 2021	Within subjects, pretest-posttest quantitative design assessing non-taste exposure	Italian parents with children between 18 and 48 months (*n* = 61 children) with access to a tablet or willing to use a tablet from the researchers	Families received a link for an app with customizable e-books about vegetables from farm to fork to read with their child daily. Parents were encouraged, but not required to offer the vegetables in the book to their child during the two-week timeframe.	An emailed pretest and posttest survey gathered demographic information, vegetable choice, parent rating of willingness to try, portion size, food frequency, liking, and book engagement (posttest only). Parents rated statements from the Children’s Eating Behavior Questionnaire: Food Fussiness Subscale and subcomponents of the Family Mealtime Goals Questionnaire.	Main effects and effect size:
					Willingness to try *F*_(1, 48)_ = 15.06, *p* < 0.001, ηp^2^ = 0.239 Portion size *F*_(1, 48)_ = 23.80, *p* < 0.001, ηp^2^ = 0.332 Consumption frequency *F*_(1, 46)_ = 8.47, *p* = 0.006, ηp^2^ = 0.156 Liking *F*_(1, 48)_ = 22.04, *p* < 0.001, ηp^2^ = 0.315
					Main effect of vegetable:
					Willingness to try *F*_(1, 48)_ = 0.01, *p* = 0.914, ηp^2^ = 0.000 Portion size *F*_(1, 48)_ = 0.00, *p* = 0.950, ηp^2^ = 0.000 Consumption frequency *F*_(1, 46)_ = 1.05, *p* = 0.310, ηp^2^ = 0.022 Liking *F*_(1, 48)_ = 0.17, *p* = 0.683, ηp^2^ = 0.004
					Interaction of vegetable and time:
					Willingness to try *F*_(1, 48)_ =0.29, *p* = 0.595, ηp^2^ = 0.006 Portion size *F*_(1, 48)_ = 0.87, *p* = 0.355, ηp^2^ = 0.018 Consumption frequency *F*_(1,46)_ = 1.25, *p* = 0.269, ηp^2^ = 0.027 Liking *F*_(1, 48)_ = 0.09, *p* = 0.767, ηp^2^ = 0.002
[17] Dazeley and Houston Price, 2015	Quasi-experimental, between-subjects design assessing non-taste exposure	Children between the ages of 12 and 36 months attending nurseries in the United Kingdom (*n* = 92)	Children were randomly assigned to the control (*n* = 37) or experimental group (*n* = 55). The experimental group was assigned to one of two sets of four foods to explore using sight, sound, smell, or touch during a four-week exposure phase while the control group had regular services.	Data were collected in a test phase by trained nursery staff to increase children’s comfort. Children were individually given food using a forced choice procedure with food from sets A and B, in a series of four plates. The order of plates was counterbalanced using a Latin square and placement of food was counterbalanced within participants. The order participants touched and tasted (put food to mouth) was recorded and verified with video.	Combined group willingness to taste exposed foods compared to non-exposed foods: t(53) = 1.65, *p* = 0.11, Combined group willingness to touch exposed foods compared to non-exposed foods: t(53) = 2.05, *p* = 0.046
[14] Dixon et al., 2023	Descriptive qualitative study with a phenomenological approach of teachers’ perspective of exposures	Head Start teachers (*n* = 35, 94% female, average age 40.8) from various geographic areas of North Carolina	Semi-structured, 45–60 min phone interviews were conducted and recorded with participants to increase understanding of teachers’ perspectives on food-based learning for nutrition curriculum and training development.	Demographic questionnaires were used following the US Office of Management and Budget protocols. Interviewers completed training and bracketing prior and interviews were transcribed. Researchers summarized responses at the end of the call and in a post-interview email for confirmation. Coding was completed by two independent researchers with Moustakas’ structured method.	11 primary themes: (1) Inside mealtime environment, (2) outside mealtime environment, (3) teacher’s use of unhealthy foods, (4) uncertainty on how to integrate food-based learning into science, (5) feelings of helplessness, (6) food waste, (7) policy, (8) COVID-19, (9) motivators for food-based learning, (10) perceptions of successful food-based learning, (11) make the connection between FBL and science to promote kindergarten readiness
[18] Izumi et al., 2015	Quasi-experimental pretest-posttest design (non-randomization of the control group) assessing taste and mixed exposures	Head Start children in five centers operated by the Mount Hood Community College Head Start program in Portland, Oregon with data analysis conducted on those with pre- and post-intervention data (*n* = 226)	The eight-month intervention with nine target fruits and vegetables was conducted to assess willingness to try and food liking. Interventions were taste only, adding target foods to menus twice weekly (low-intervention), or taste combined with nutrition education using the Harvest for Healthy Kids curriculum (high-intervention) with mealtime conversation starters, books, cooking, crafts, tasting, and newsletters.	Demographic information was collected from Head Start enrollment data. Trained researchers assessed each child’s willingness to try and food liking. Willingness to try was recorded dichotomously as 1, tried the sample, or 0, refused. Liking was assessed based on the method developed by Birch using a nonverbal hedonic scale with a smiling face indicating “I like it”, a neutral face depicting “It’s okay”, and a frowning face showing “I don’t like it yet”.	Low intervention (taste exposure) willingness to try, compared to control:
					Turnip OR = 3.5, 95% CI [1.5–9.2] Rutabaga OR = 3.9, 95% CI [1.7–9.1] Beet OR = 2.4, 95% CI [1.1–5.4]
					High intervention (mixed exposure) willingness to try, compared to control:
					Carrot OR = 5.7, CI [1.4–23.0] Butternut squash OR = 2.9, CI [1.1–7.4] Sweet potato OR = 4.3, CI [1.8–10.7] Turnip OR = 7.2, CI [2.7–10.5] Rutabaga OR = 9.6, CI [3.8–24.5] Beet OR = 6.1, CI [2.3–16.3]
					Low intervention (taste exposure) liking, compared to control:
					No statistically significant results
					High intervention (mixed exposure) liking, compared to control:
					Sweet potato OR = 3.4, 95% CI [1.2–9.5] Turnip OR = 3.7, 95% CI [1.2–11.2] Berries OR = 21.2, 95% CI [2.4–187.3]
					No statistical differences between intervention willingness to try or liking.
[19] Johnson et al., 2019	Longitudinal, quasi-experimental design (Colorado Longitudinal Eating and Physical Activity (LEAP) study) for mixed exposures	Children in Colorado preschool centers, including Head Start, in two geographically and economically diverse areas who were advancing to Kindergarten next year (*n* = 250), data from those with special dietary needs and developmental disabilities were excluded from analysis	A 12-week program, Food Friends—Fun with New Foods^®^, was implemented in intervention classrooms by trained teachers with classroom-based exposures of nine foods with additional materials sent home to caregivers. Targeted foods included garbanzo beans, grapefruit, Gouda cheese, couscous, spinach, salmon, beets, pineapple, and jicama. Jicama was one target food with tasting opportunities once a week for the first eight weeks, the second was edamame, assessed without taste exposures. The control groups did not implement the program and had regularly scheduled lessons. In the two years of follow-up, monthly booster lessons were implemented during kindergarten and first grade, with support materials sent home.	A demographic questionnaire was sent home and completed by parents. Height and weight were measured by trained researchers using a digital scale and portable stadiometer. To evaluate program fidelity, a teacher survey was completed on activity preference and child engagement. Liking was measured with the preference assessment developed by Sullivan and Birch, using a nonverbal hedonic scale to rate as “Yummy”, “Just Okay”, and “Yucky”. Consumption was measured based on pre-and post-meal weight of food.	Intervention jicama consumption:
					Baseline mean = 16.7 g, CI [10.9–22.5] Post-intervention mean = 34.1 g, CI [28.2–40.0]; change from baseline mean = 17.4 (12.3, 22.5), *p* < 0.0001 Two-year follow-up mean = 37.3 g, CI [30.8–43.8]; change from baseline mean = 20.6 (14.8, 26.4), *p* < 0.0001
					Comparison jicama consumption:
					Baseline mean = 7.3 g, CI [0.7–14.0] Post-intervention mean = 10.6 g, CI [4.1–17.2]; change from baseline mean = 3.3 (−3.1, 9.7), *p* = 0.3094 Two-year follow-up mean = 20.3 g, CI [12.6–28], change from baseline mean = 13.0 (5.4, 20.5), *p* = 0.0008
					Intervention edamame consumption:
					Baseline mean = 7.6 g, CI [−0.2–15.5] Post-intervention mean = 12.4 g, CI [4.5–20.4]; change from baseline mean = 4.8 (0.5, 9.1), *p* = 0.0280 Two-year follow-up mean = 20.8 g, CI [12.6–29.1]; change from baseline mean = 13.2 (8.0, 18.4), *p* < 0.0001
					Comparison edamame consumption:
					Baseline mean = 7.5 g, CI [−0.9–15.8] Post-intervention mean= 9.5 g, CI [1.2, 17.7]; change from baseline mean = 2.0 (−3.4, 7.4), *p* = 0.4652 Two-year follow-up mean = 28.9 g, CI [19.9–37]; change from baseline mean = 21.4 (14.6, 28.2), *p* < 0.0001
					Significant difference in the jicama consumption over time between intervention and control: *p* < 0.005
					Intervention group consumption of those rating jicama as “yummy” at baseline:
					Post-intervention change from baseline mean = 18.6 g CI [11.0–26.3], *p* < 0.0001 Two-year follow up change from baseline mean = 21.4 g CI [13.4–29.5], *p* < 0.0001
					Intervention Liking:
					Baseline rating yummy = 54.7% Post-intervention rating yummy = 73.9%; significant change from baseline p < 0.05 Two-year follow up rating yummy = 77.4%; significant change from baseline *p* < 0.001
					Control Liking:
					Baseline rating yummy = 39.1% Post-intervention rating yummy = 44.3%; significant change from baseline *p* < 0.05 Two-year follow up rating yummy = 50.8%; significant change from baseline *p* < 0.001
					Significant change over time for both groups, *p* = 0.0002; Liking differences not statistically significant between groups, *p* = 0.1980
[20] Masento et al., 2023	Within subjects, pretest-posttest quantitative design for non-taste exposure	United Kingdom families with children between the ages of 18 and 48 months and access to a tablet device (*n* = 36)	Parents received links to the *See & Eat* books on the Our Story 2 application with the customizable story focusing on the vegetable journey from farm to fork. Parents were asked to select two vegetables that their child does not typically eat for assessment.	Demographics were collected via a preintervention questionnaire. This questionnaire also contained questions on parent feeding style and child eating behavior. After the target and control vegetables were selected, parents rated their child’s willingness to try, intake, and liking on Likert scales, some from adapted versions of the Child Food Frequency Questionnaire and Fruit and Vegetable Familiarity and Liking Questionnaire.	Main effect of condition:
					Willingness to try *F*_(1, 29)_ = 5.10, *p* = 0.032, ηp^2^ = 0.15 Portion size *F*_(1, 29)_ = 4.62, *p* = 0.04, ηp^2^ = 0.14 Consumption frequency *F*_(1, 30)_ = 7.55, *p* = 0.01, ηp^2^ = 0.201 Liking *F*_(1, 29)_ = 5.98, *p* = 0.021, ηp^2^ = 0.171
					Main effect of time:
					Willingness to try *F*_(1, 29)_ = 12.12, *p* = 0.002, ηp^2^ = 0.30 Portion size *F*_(1, 29)_ = 29.13, *p* < 0.001, ηp^2^ = 0.50 Consumption frequency *F*_(1, 30)_ = 19.25, *p* < 0.001, ηp^2^ = 0.391 Liking *F*_(1, 29)_ = 23.54, *p* < 0.001, ηp^2^ = 0.448
					Interaction effects between the condition and time:
					Willingness to try *F*_(1, 29)_ = 2.93, *p* = 0.098, ηp^2^ = 0.092 Portion size *F*_(1, 29)_ = 15.55, *p* < 0.001, ηp^2^ = 0.349 Consumption frequency F_(1, 30)_ = 13.49, *p* = 0.001, ηp^2^ = 0.31 Liking *F*_(1, 29)_ = 8.69, *p* < 0.05, ηp^2^ = 0.231
[21] Monsur et al., 2024	Randomized two-group, pre-post experimental design, non-taste exposure	Three- to five-year-old children from eight Texas childcare centers participated with parent consent (*n* = 149)	Raised garden beds and a garden activity book were implemented in experimental centers with six vegetables (cucumbers, green beans, bell peppers, tomatoes, yellow squash, and zucchini) and five fruits (blackberries, blueberries, cantaloupe, strawberries, and watermelon) planted. The garden activity guide included planting, maintenance, and harvesting.	In an individual interview at pre- and post-intervention, children’s knowledge and liking of fruits and vegetables were measured. Liking was assessed on a five-point face scale, ranging from super yucky to super yummy, and knowledge was assessed with food identification. The interviewer placed single fruits and vegetables on a table for identification, and the session was recorded. A garden activity chart was completed for each child’s participation in the activities.	Fruit liking:
					Experimental pre-intervention = 2.39 Experimental post-intervention = 2.36 Control pre-intervention = 2.72 Control post-intervention = 2.37 Estimated effect = 0.92, *p* = 0.19
					Vegetable liking:
					Experimental pre-intervention = 2.87 Experimental post-intervention = 3.18 Control pre-intervention = 3.33 Control post-intervention = 2.00 Estimated effect = 2.05, *p* = 0.04
					Fruit and vegetable liking
					Experimental pre-intervention = 2.48 Experimental post-intervention = 2.84 Control pre-intervention = 2.85 Control post-intervention = 2.25 Estimated effect = 1.20, *p* = 0.01
[22] Nekitsing et al., 2019	Cluster randomized control trial using a 2 × 2 factorial parallel design assessing taste, non-taste, and mixed exposures	Two- to five-year-old children attending 11 preschools in the United Kingdom	Mooli was selected as the intervention target vegetable. A 2 × 2 factorial parallel design was used for cluster, stratified randomization and the four groups received weekly taste exposure, nutrition education, nutrition education with taste exposure, or no intervention for 10 weeks. The Phunky Foods nutrition education program was used; however, mooli was not part of this program.	Mooli intake was measured in grams (g) at week 1, week 12, and at two follow-up points, weeks 24 and 36. Prepared and weighed snack bags of mooli were given at snack time and researchers measured post-consumption weight.	No Intervention:
					Baseline mean intake = 3.5 ± 2.7 g, 25% unwilling to try Post-intervention mean intake = 6.1 ± 2.8 g, 25% unwilling to try 24-week mean intake = 9.5 ± 4.6 g, 31.2% unwilling to try 36-week mean intake = 10.3 ± 3.9 g, 25% unwilling to try
					Taste Exposure:
					Baseline mean intake = 4.7 ± 1.4 g, 31.9% unwilling to try Post-intervention mean intake = 17.0 ± 2.7 g, 6.4% unwilling to try 24-week mean intake = 17.9 ± 2.7 g, 17% unwilling to try 36-week mean intake = 20.1 ± 2.5 g, 17% unwilling to try Eaters χ^2^[1] = 4.67, *p* = 0.031 Increased intake of those who were eaters: *F*_(1,135)_= 11.21, *p* = 0.001
					Nutrition Education (non-taste):
					Baseline mean intake = 5.5 ± 1.8 g, 18.4% unwilling to try Post-intervention mean intake= 8.0 ± 1.7 g, 0% unwilling to try 24-week mean intake = 11.5 ± 2.1 g, 7.9% unwilling to try 36-week mean intake = 17.6 ± 2.8 g, 10.5% unwilling to try Intake of those who were eaters: *F*_(1, 135)_ = 0.47, *p* = 0.49
					Nutrition Education with Taste (Mixed):
					Baseline mean intake = 11.0 ± 2.9 g, 35.9% unwilling to try Post-intervention mean intake = 17.8 ± 3.1 g, 10.3% unwilling to try 24-week mean intake = 23.9 ± 4.0 g, 15.4% unwilling to try 36-week mean intake = 20.8 ± 2.9, 10.3% unwilling to try
					Odds of being an eater (willing to try):
					Interaction between taste exposure and nutrition education: χ^2^[1] = 4.67, *p* = 0.031 Nutrition education conditions (mixed and non-taste groups): χ^2^[1] = 5.73, *p* = 0.017 Taste Exposure odds of eating mooli: χ^2^[1] = 0.24, *p* = 0.63 No main effect of time: χ^2^[1] = 5.82, *p* = 0.054
[15] Roberts et al., 2022	Experimental, randomized control study, non-taste exposure	Children from nurseries in south-east England between the ages of 36 to 59 months (*n* = 110)	The study assessed intake and willingness to try after combinations of visual, touch, and smell exposures of six vegetables (broccoli, fennel, leek, parsnip, radish, and swede). Visuals were controlled using a covering of black material, and a mesh cloth with a lid was used to control smell. Matching games with food and colored posters were used. The control received six toys using the same methods. Exposures were assessed as visual only, smell-visual, and smell-visual-tactile.	All children received two pieces of each food. Willingness to try was measured by awarding a point for each food touched to their tongue or lips, with a total of six possible (one for each vegetable). Intake was measured on a two-point scale, with one point awarded for each piece of the vegetable eaten (two pieces of each food), for a total of 12 points possible.	Willingness to try:
					Visual compared to control: U = 328.0, *p* = 0.286, η^2^ = 0.02 Smell-visual compared to control: U = 174.0, *p* < 0.001, η^2^ = 0.21 Smell-visual-tactile compared to control: U = 171.0, *p* < 0.001, η^2^ = 0.26 Smell-visual-tactile compared to visual-only: U = 252.5, *p* = 0.012, η^2^ = 0.12 Smell-visual compared to visual-only: U = 265.0, *p* = 0.069, η^2^ = 0.06 Smell-visual compared to smell-visual-tactile: U = 321.5, *p* = 0.394, η^2^ = 0.01
					Intake:
					Visual-only compared to control: U = 388.5, *p* = 0.954, η^2^ < 0.001 Smell-visual compared to control: U = 222.0, *p* = 0.014, η^2^ = 0.11 Smell-visual-tactile compared to control: U = 193.0, *p* = 0.001, η^2^ = 0.19 Smell-visual compared to visual-only: U = 247.5, *p* = 0.043, η^2^ = 0.08 Smell-visual compared to smell-visual-touch: U = 309.0 *p* = 0.339, η^2^ = 0.02 Smell-visual-tactile compared to visual-only: U = 212.0, *p* < 0.003, η^2^ = 0.16
[7] Rubio and Rigal, 2017	Qualitative study using focus groups of caregivers.	French parents of children aged 18 to 36 months in childcare centers (*n* = 38) with self-reported low or moderate income.	A total of six focus groups were completed to understand children’s food pickiness, including onset and affected foods, parent perceptions, and strategies for pickiness.	Focus groups included a moderator leading the discussion and an assistant recording and taking notes. The sessions were 65 to 80 min, ending when no additional information emerged. A discussion guide was developed and tested using a pilot group. A researcher and assistant independently completed a thematic analysis, reaching agreement on themes.	6 Themes: (1) changes in food behaviors, (2) type of food affected by changes, (3) manifestations of pickiness, (4) attributions of toddler’s pickiness, (5) consequences of food pickiness, (6) parental strategies to overcome pickiness
[23] Schmitt et al., 2020	Quasi -experimental, pilot study, mixed exposures	Children in two Head Start centers (*n* = 39) with the majority male (*n* = 25) and Caucasian (*n* = 31)	A five-week intervention with 15 short lessons was implemented to assess self-regulation and liking of fruits and vegetables with pre-and post-intervention tests. Lessons included classroom-based activities, such as mindfulness, memory or physical activity games, and food preparation, many using real fruits and vegetables. Children in the comparison received regular services.	The Head–Toes–Knees–Shoulders Task (HTKS) measured behavioral regulation. The National Institutes of Health (NIH) Toolbox Flanker Inhibitory Control and Attention Test and Toolbox Dimensional Change Card Sort Test measured executive function. Children rated food as “Yucky”, “Just Okay”, and “Yummy” to assess food liking. Teacher surveys and researcher observation measured program fidelity.	Experimental, vegetable liking mean change = 1.1, *p* = 0.02 Comparison, vegetable liking mean change = 0.3, *p* = 0.45
[24] Smith et al., 2020	Experimental, randomized controlled design, mixed exposures	Head Start children at a rural Ohio center without asthma or diabetes (*n* = 209)	In three randomly assigned and demographically similar groups, researchers studied if nutrition education and access impacted consumption after an eight-week intervention. A comparison classroom received regular services, treatment A classroom received high-carotenoid fruits and vegetables weekly, and treatment B classroom received weekly food and nutrition education from the Harvest for Healthy Kids program.	Blood carotenoid levels were assessed before and after the intervention using a Pharmanex BioPhotonic S3 Scanner using Resonance Ramen Spectroscopy. This uses light for measurement and has been validated with serum levels.	Comparison:
					Change score = 2623 Raman units (RU)
					Treatment A (high-carotenoid fruits and vegetables weekly, taste exposure):
					Change score = 4887 RU Comparison vs. treatment A *p* = 0.10
					Treatment B (weekly food and nutrition education, mixed exposure):
					Change score = 7834 RU Comparison vs. treatment B *p* < 0.001 Treatment A vs. B *p* < 0.02
					Difference in carotenoid scores of treatments = *F*2, 206 = 12.967, *p* < 0.001, Cohen D = 71
[25] Swindle et al., 2020	Non-randomized, within-subjects, quasi-experimental design for home-effect of mixed exposures	Parents of enrolled children at seven Head Start centers in Louisiana and Arkansas	This study assessed the Together, We Inspire Smart Eating (WISE) program to determine if classroom interventions impact parental decisions at home through pester power. Windy Wise is the barn owl mascot of the program, encouraging and teaching children about food and nutrition. The curriculum provides a variety of opportunities for sensory explorations of food, including taste and non-taste exposures, and caregiver materials.	Caregivers were interviewed by trained researchers pre- and post-intervention to evaluate consumption and willingness to try WISE foods and nutrient-poor foods. Parents were asked to complete an adapted food frequency questionnaire from the Family Map Inventory tool used in some Head Start programs. Parenting feeding practices were measured using an adapted version of the Fruit and Vegetable Parenting Practices Questionnaire. Child acceptance of new foods, including willingness to try, was measured using aspects of the Toddler-Parent Mealtime Behavior Questionnaire. Questions were developed to measure pester power post-implementation.	Pretest WISE food mean intake = 3.0 ± 0.90 Posttest WISE food mean intake = 3.4 ± 0.69 Correlation of pretest and posttest WISE food intake: r = 0.54, *p* < 0.01 Correlation of pester power and posttest WISE fruit and vegetable intake: r = 0.31, *p* < 0.01 Pester power as a predictor of WISE food intake: B = 0.16, β = 0.29, *p* < 0.01
[26] Whiteside-Mansell & Swindle, 2019	Quasi-experimental pretest-posttest design, mixed exposures	Parents of children enrolled in 15 Head Start centers in a rural, southern state (*n* = 526)	The WISE program was assessed by comparing parent-reported consumption in a pre/post format. Educators were trained in food preparation experiences and given ideas for integrating nutrition into educational activities. Additional information was shared via Facebook.	Family map inventories, a tool used in some Head Start programs, were completed in semi-structured pre- and post-intervention interviews with parents. The inventory included an adapted food frequency questionnaire focusing on the 10 WISE foods (apples, tomatoes, sweet potatoes, strawberries, carrots, bell peppers, spinach, greens, green beans, and blueberries) and other foods consumed in the home.	Weekly consumption of dark green, orange, and yellow vegetables:
					Comparison baseline mean = 5.93 ± 3.19; follow-up mean = 6.10 ± 3.07 WISE baseline mean = 5.74 ± 3.11; follow-up mean = 6.54 ± 2.96 Regression model estimates using FMIL = 0.11 (SE = 0.050, t = 2.12, *p* < 0.05)
					Weekly consumption of fruits like apples, oranges, bananas, grapes, peaches:
					Comparison baseline mean = 7.81 ± 2.34; follow-up mean = 8.10 ± 2.08 WISE baseline mean = 7.70 ± 2.48; follow-up mean = 8.27 ± 1.96 Regression model estimates using FMIL = 0.05 (SE = 0.058, t = 0.79)
					Weekly consumption of sugary sweets like cakes and candy, or sugary drinks:
					Comparison baseline mean = 4.22 ± 2.90; follow-up mean = 4.36 ± 2.48 WISE baseline mean = 4.22 ± 2.62; follow-up mean = 3.71 ± 2.61 Regression model estimates using FMIL = −0.16 (SE = 0.050, t = −3.27, *p* < 0.01)
					Monthly consumption of WISE vegetables:
					Comparison baseline mean = 4.73 ± 6.54; follow-up mean = 4.82 ± 7.54 WISE baseline mean = 3.26 ± 3.45; follow-up mean = 3.73 ± 4.10 Regression model estimates using FMIL = 0.02 (SE = 0.044, t = 0.49)
					Monthly consumption of WISE fruits:
					Comparison baseline mean = 9.99 ± 11.63; follow-up mean = 8.63 ± 10.19 WISE baseline mean = 7.35 ± 7.22; follow-up mean = 8.48 ± 7.17 Regression model estimates using FMIL = 0.12 (SE = 0.050, t = 2.12, *p* < 0.05)

**Table 3:** Depiction of taste, non-taste, and mixed food exposures on children’s willingness to try, consume, and like foods.

	Taste	Non-Taste	Mixed
Willingness to Try	↑ [18], ↔ [22]	↑ [15], ↔ [17], ↑ [8], ↑ [20]	↑ [18]
Consumption	↑ [22], ↔ [24]	↑ [15], ↔ [22], ↑ [8], ↑ [20]	↑ [24], ↑ [19], ↑ [26], ↑ [25]
Like	↔ [18]	↑ [20], ↑ [21]	↑ [18], ↑ [19], ↑ [23]

↔ No significant effect of exposure relative to baseline or control group; ↑ Significantly improved in exposure group relative to baseline and/or control group.
